# Poisonous Action of the Aqueous Vapor of Expired Air

**Published:** 1896-02

**Authors:** 


### Poisonous Action of the Aqueous Vapor of Expired Air.

Dr. Livierato (*Arch. Ital. di Biol.*, 1895, p. 279) condensed the aqueous vapor of the breath of persons afflicted with disease of the respiratory tract accompanied by fever, of the same without fever, of persons with fever but with no respiratory disease, and of persons in health, and injected the liquids obtained into rabbits. That from the first mentioned caused a fever lasting from three to six days, dullness, and diminished reflexes; that from the patients with no fever gave the same results, but less marked; that from persons with fever but no respiratory disease produced little or no effect, and that from persons in health none whatever.—*American Journal Med. Sciences.*

